# Cerebral Blood Flow Measurement Using fMRI and PET: A Cross-Validation Study

**DOI:** 10.1155/2008/516359

**Published:** 2008-09-24

**Authors:** Jean J. Chen, Marguerite Wieckowska, Ernst Meyer, G. Bruce Pike

**Affiliations:** McConnell Brain Imaging Centre, Montreal Neurological Institute, McGill University, Montreal, PQ, Canada H3A 2B4

## Abstract

An important aspect of functional magnetic resonance imaging (fMRI) is the study of brain hemodynamics, and MR arterial spin labeling (ASL) perfusion imaging has gained wide acceptance as a robust and noninvasive technique. However, the cerebral blood flow (CBF) measurements obtained with ASL fMRI have not been fully validated, particularly during global CBF modulations. We present a comparison of cerebral blood flow changes (ΔCBF) measured using a flow-sensitive alternating inversion recovery (FAIR) ASL perfusion method to those obtained using H_2_^15^O PET, which is the current gold standard for in vivo imaging of CBF. To study regional and global CBF changes, a group of 10 healthy volunteers were imaged under identical experimental conditions during presentation of 5 levels of visual stimulation and one level of hypercapnia. The CBF changes were compared using 3 types of region-of-interest (ROI) masks. FAIR measurements of CBF changes were found to be slightly lower than those measured with PET (average ΔCBF of 21.5 ± 8.2% for FAIR versus 28.2 ± 12.8% for PET at maximum stimulation intensity). Nonetheless, there was a strong correlation between measurements of the two modalities. Finally, a *t*-test comparison of the slopes of the linear fits of PET versus ASL ΔCBF for all 3 ROI types indicated no significant difference from unity (*P* > .05).

## 1. INTRODUCTION

Neuronal activity results in focal changes in hemodynamics, metabolism, and blood oxygenation of associated brain areas. Functional maps of cerebral blood flow (CBF) can be used to monitor hemodynamic changes in the healthy brain as well as alterations associated with cerebrovascular disease. Positron emission tomography (PET) is capable of providing in vivo quantitative measures of CBF and has evolved to be considered the gold standard for studying cerebral hemodynamics. However, PET imaging involves the injection of radioactive tracers, which limits its repeatability and application in healthy volunteers. Among other limitations are low temporal and spatial resolution, low signal-to-noise ratio (SNR), as well as the requirement for a cyclotron. Thus, magnetic resonance (MR) perfusion imaging, being widely available and having relatively high spatial and temporal resolution, is increasingly seen as an attractive alternative to PET.

MR perfusion imaging is performed using dynamic susceptibility contrast (DSC) techniques or arterial spin labeling (ASL) [1, 2]. DSC imaging has not been widely applied in human functional research due to the requirement of an exogenous contrast agent and limited temporal resolution. ASL is based on the detection of magnetically labeled arterial blood water spins and has therefore been used with more success in functional MRI (fMRI) studies. Pulsed ASL methods such as proximal inversion with control for off-resonance effects (PICORE), flow-sensitive alternating inversion recovery (FAIR), quantitative imaging of perfusion using a single subtraction (QUIPSS I/II), and QUIPSS II with thin-slice TI1 and periodic saturation (Q2TIPS) have greatly facilitated perfusion-based fMRI [2–8].

The validation of MR perfusion measurements using various invasive and noninvasive methods has been a topic of considerable interest. Walsh et al. compared CBF measured using continuous ASL and radioactive microspheres using a rat model and found ASL to underestimate CBF under high flow [9]. On the other hand, based on radiotracer-enhanced quantitative autoradiography flow measurements in rats, Ewing et al. concluded that CBF were overestimated by ASL under ischemia [10]. In healthy humans, Østergaard et al. [11] found a highly linear relationship between PET and DSC MR CBF measurements (using Gd-DTPA), consistent with the values reported in the literature. Similar findings were reported by Carroll et al. [12], Lin et al. [13], and Grandin et al. [14] in healthy human subjects. Quantitative CBF values of 68.1 ± 9.5 and 26.7 ± 5.0 mL/100 g/min were measured for grey matter (GM) and white matter (WM), respectively [13]. In addition, Grandin et al. reported a high correlation between DSC and PET CBF measurements under the effect of vasodilative pharmacological agents, with PET results having higher reproducibility [14]. However, Carroll and Grandin found that GM CBF values were overestimated with DSC MR possibly due to sensitivity to the presence of large blood vessels [12, 14].

Ye et al. reported a comparison of resting CBF using steady-state ASL and PET and measured GM CBF of 64.12 and 67.13 mL/100 g/min, respectively. The PET and ASL measurements were not statistically different from one another and were both in good agreement with literature values [15]. However, the WM ASL CBF (23.8 mL/100 g/min) was 30% lower compared to PET (33.7 mL/100 g/min), the discrepancy being attributed to the arterial tagging time difference between GM and WM, specific to the quantitative model employed in this study. In epilepsy patients, Liu et al. studied perfusion in the temporal lobe using the FAIR-prepared half-Fourier single-shot turbo spin-echo (HASTE) technique [16] and also found a statistically significant correlation between ASL and PET data. A functional comparison involving PET and ASL was first performed by Zaini et al. using a simple finger-tapping task [17]. However, the matching of spatial resolution and noise was not possible for PET and ASL data. In a more recent study by Feng et al. [18], another comparison of PET and FAIR fMRI measurements of CBF changes was reported, using a single level of visual stimulation in healthy subjects. Once again, results obtained by the two methods were very similar, with the PET CBF percent change being slightly higher than that of FAIR (ΔCBF of 38.79% versus 36.95%).

Notwithstanding the contributions of the above studies, several factors limit the scope and applicability of the existing studies. Firstly, an accurate comparison of PET and fMRI perfusion is challenging due to methodological differences. In particular, spatial resolution disparities lead to difficulties in accurate region-of-interest (ROI) registration and partial-volume matching, which are critical for direct comparisons. Secondly, both techniques are inherently sensitive to physiological variations, which reduce measurement reproducibility. Carroll et al. measured interexam ASL CBF variation in a single subject to be as high as 20 mL/100 g/min in GM and 15 mL/100 g/min in WM, while those observed with PET were 4-5 mL/100 g/min [12]. Grandin et al. observed variations of up to 13% for PET and 16% for MR CBF measurements at rest in the same individual [14]. As a result, high intersubject and interexam variability between PET and MR are expected, particularly in inexperienced volunteers scanned over several days. Thirdly, past comparisons of CBF measurements were largely performed without functional stimulation, and for those within the fMRI context, the impact of graded stimulus intensity [8] has not been explored. Finally, previous ASL and PET CBF data were not always collected in similar environments.

We were interested in evaluating the relative accuracy of the FAIR ASL method for ΔCBF measurements in comparison with PET. Previous ASL research [19, 20] has shown the accuracy of FAIR in determining CBF to be dependent on the transit delay and label width, which can be variable across subjects and experimental conditions. This has led to the introduction of techniques less sensitive to transit delay and bolus width, such as QUIPSS II [4], and their adoption by our group [21–23] and others. However, the FAIR technique [24] has been and continues to be used extensively in the literature as a well-established method for the investigation of functional hemodynamics [3, 6–8]. Notably, some widely adopted and investigated biophysical models of the BOLD signal have been developed and validated based on CBF data using FAIR [3, 18, 25–27]. Thus, a dedicated assessment of the validity of FAIR for CBF measurement would be a highly valuable addition to our knowledge.

In this study, we compare FAIR fMRI measurements of CBF with those made using PET during graded levels of visual stimulation. In addition, we measured CBF changes induced by hypercapnia, which has been employed to explore global, activation-independent perfusion increases and applied to cross-subject calibrations of the BOLD response [25]. Also, over the course of our experiments, the conditions for ASL and PET data collection were also closely matched and monitored.

## 2. METHODS AND MATERIALS

### 2.1. Experimental design

Visual stimuli were generated using locally developed software (GLStim) based on the OpenGL graphics library (Silicon Graphics, Mountain View, Calif, USA). The baseline condition consisted of a uniform grey field, while the activation pattern was a yellow-blue radial checkerboard with 30 spokes and 6.5 rings of equal radial thickness, reversing contrast at 4 Hz. The checkerboard contains both color and luminance contrast designed to produce robust local CBF increase in the primary visual cortex (V1) [3, 8]. In an effort to maintain the subjects' attention, a fixation task (a small arrow randomly changing directions) was present at the centre of the field of view (FOV) throughout the scans. Subjects were requested to continuously report the arrow direction by means of an MR-compatible mouse.

**Figure 1:**
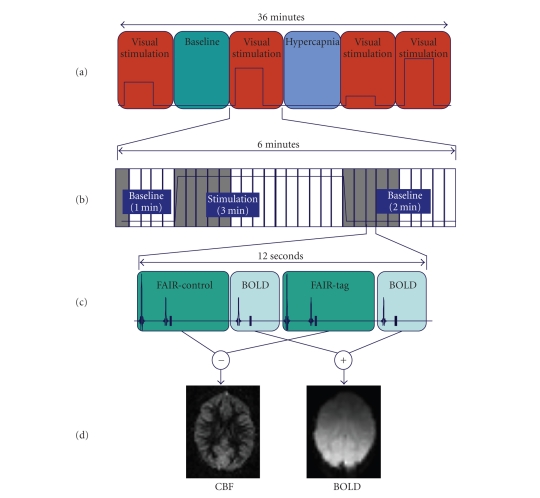
(a) The fMRI experimental protocol consisted of 6 randomly presented sessions (baseline, hypercapnia, and 4 levels of visual stimulation). (b) The interleaved BOLD-FAIR sequence was repeated 30 times during each run of 6 minutes, composed of a 1-minute baseline, 3-minute stimulation, and 2-minute baseline period. Scans shaded in grey (1 minute post onset and cessation of stimulation plus first scan in the run) were excluded in percent change calculations to ensure that time-averaged data included only physiological changes in steady state. (c) The basic structure of the BOLD-FAIR sequence consists of 2 BOLD acquisitions (averaged to form 1 BOLD-contrast image) interleaved with 1 slice selective and 1 nonselective ASL acquisition. (d) These 4 acquisitions produce a flow-weighted FAIR image through subtraction and a BOLD image through addition.

**Figure 2:**
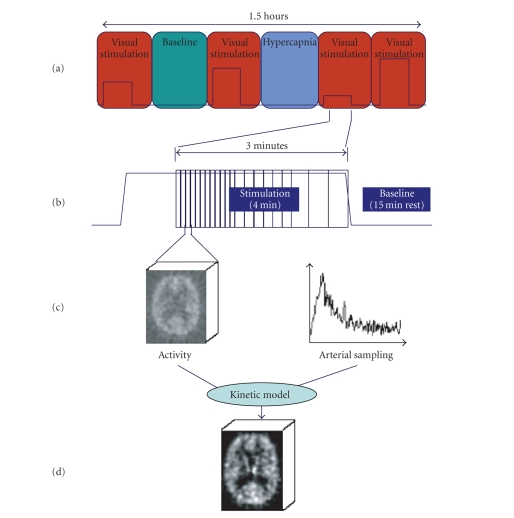
(a) The PET experimental protocol consisted of 6 randomly presented sessions (baseline, hypercapnia, and 4 levels of visual stimulation). A tracer bolus injection was given at the start of each block. (b) Each run has a 3-minute acquisition period divided in 12 5-second, 6 10-second, and 3 20-second frames, followed by 15 minutes of rest. (c) A volume of activity distribution was acquired during each frame. (d) This series of distribution images and the blood activity curve were fit into a kinetic model, resulting in a CBF map.

The graded visual stimulation and hypercapnia schemes were matched to those previously employed in calibrated fMRI studies of flow-metabolism coupling [3]. In addition to the uniform grey-field reference condition, the subjects were presented with 4 graded levels of visual stimulation, ranging from 25% to 100% intensity, while inhaling atmospheric composition medical air supplied at 16 L/min. Furthermore, mild hypercapnia (induced using air mixture of 5:21:74% CO_2_:O_2_:N_2_) was used to study global CBF changes. Both PET and ASL scans included 6 sessions of 3 minutes, each consisting of one visual-respiratory condition played out continuously. Each stimulation session was preceded and followed by a baseline condition (Figures 1 and 2) of 1 and 2 minutes, respectively.

A total of 10 healthy human subjects (8 males, 2 females), aged 23.9 ± 3.3 years, were imaged under the above six experimental conditions. Informed consent was obtained from every subject prior to each PET and MRI scanning session, with the experimental protocol being approved by the Research Ethics Board of the Montreal Neurological Institute (MNI, Montreal, Canada). In order to achieve maximal similarity between the PET and fMRI experimental conditions, the sizes of the projected checkerboards were matched, as well as the lighting intensity at the two imaging locations. During the scans, subjects were asked to breathe through a nonrebreathing face mask, allowing control of the incoming air composition.

### 2.2. Magnetic resonance imaging

Subjects were immobilized with a foam headrest and head restraints. A nasal cannula connected to a capnometer was used to monitor end-tidal carbon dioxide (ETCO_2_) and the respiratory rate, whereas the arterial oxygen saturation (O_2_Sat) and the pulse rate were measured with a finger pulse oximeter. The pulse and respiratory rates are indicators of blood CO_2_ tension. The stimulus was presented by an adjustable back-projection mirror mounted on the head coil.

The MR scans were performed on a 1.5 T Siemens Magnetom Vision system (Siemens, Erlangen, Germany). To optimize the SNR of the functional data in the visual cortex, a transmit-receive surface coil placed near the occipital lobe was used to acquire all functional images. Thus, prior to the functional scans, a surface-coil T1-weighted (T1W) anatomical scan, acquired at a resolution of 1 × 1 × 2 mm^3^, was used in slice selection and alignment of all functional data. However, as the surface-coil anatomical data has highly nonuniform intensity, registration to PET data using our local software was difficult. Therefore, an additional high-resolution 1 × 1 × 1 mm^3^ T1W anatomical scan with a head coil was also acquired to facilitate the registration of PET and surface-coil MR data. We use the interleaved FAIR-BOLD echo-planar imaging sequence as implemented by Hoge et al. [3] in order to directly evaluate their measurements as well as to achieve simultaneous BOLD monitoring. Furthermore, we selected imaging parameters to best enable replication of experimental conditions in previous fMRI flow-metabolism studies [3, 28]. The FAIR inversion time and echo time (TE) were 900 milliseconds and 20 milliseconds, respectively, while the BOLD TE was 50 milliseconds. A 7-mm thick single oblique slice parallel to the calcarine sulcus was acquired on a 64 × 64 matrix with a 5 × 5 mm^2^ inplane voxel size. As seen in Figure 1, the repetition time of the sequence was 12 seconds, allowing acquisition of a total of 60 frames per 6 minutes run, 30 FAIR and 30 BOLD frames. Of these 30 frames, 11 were excluded (1 minute postonset and postcessation of stimulation plus the first scan in the run) to ensure that only data corresponding to the physiological steady-state response was examined. The chosen FAIR implementation minimizes errors related to the tagging slab arrival time and width through the use of a single-slice acquisition and a body coil inversion [8]. In order to obtain an accurate masking location of V1 (primary visual cortex), BOLD-based retinotopic mapping was performed in a separate session, using a visual stimulus composed of a thick black-and-white expanding ring, also designed using GLStim [3, 28]. A total of 16 slices of 4 mm parallel to the calcarine sulcus were acquired using a BOLD sequence during 6 randomly ordered runs of a 6-minute visual stimulation.

### 2.3. Positron emission tomography

The protocol for the PET experiments was adapted from previous PET studies aimed at reproducing MRI results [29]. The subjects were immobilized using a self-inflating foam headrest, which minimized motion during scans. The stimulus was presented through an adjustable mirror mounted on the patient table. As previously described, the O_2_Sat level and pulse rate were monitored using a finger pulse-oxymeter, while a capnometer connected via a nasal cannula monitored the ETCO_2_ and the respiratory rate. In addition, a short indwelling catheter was placed by an anesthetist into the left radial artery for blood sampling and a more precise examination of blood gases. A three-way stop cock allowed for simultaneous automatic (using a locally developed sampling system for blood activity measurement) and manual (for blood gases examination) blood sample withdrawal. Automatic blood sampling was performed at 0.5-second intervals throughout the data collection period. A fine needle catheter was placed into the antecubital vein of the right arm for injection of the isotope.

PET images were acquired on an ECAT EXACT HR^+^ (CTI/Siemens, Knoxville, TN) whole-body tomography system operating in three-dimensional (3D) mode. The volumetric images were reconstructed on 128 × 128 matrices of 2 × 2 mm^2^ pixels using filtered back-projection with an 8-mm Hanning filter. For each of the 6 sessions, 10 mCi of H_2_^15^O were injected. The H_2_^15^O isotopes were prepared in a Cyclone 18/9 cyclotron (IBA, Louvain-la-Neuve, Belgium) adjacent to the scanner. The reconstructed images were automatically corrected for random and scattered events, detector efficiency variation and dead time [30, 31]. Also, a transmission scan was collected for each subject before the experiments for estimating attenuation of the 511 keV gamma rays as a function of tissue density [32]. A normalization scan was acquired for eliminating effects due to ring geometry and crystal sensitivity [33]. The stimulation conditions were presented in random order. As shown in Figure 2, during each 3-minute scan, the subjects were presented with only one type of stimulation, which started 1 minute before the start of the scan. This delay was designed to enable condition matching with fMRI. Arterial blood sampling and dynamic imaging started at injection time, and each scan was followed by a 15-minute resting period, allowing the radioisotope to decay before a new injection. Due to the short half-life of H_2_^15^O (2 minutes) and its kinetic behaviour, the observed H_2_^15^O activity changes are very fast immediately after injection, requiring the acquisition of more frames at the beginning of the scan. Thus, each 3-minute scan consists of 21 frames acquired in 12 5-second intervals, followed by 6 frames at 10-second and 3 frames at 20-second intervals. Finally, due to the tracer kinetic model fitting required for PET data, only one volumetric CBF image was obtained for each experimental condition and no time evolution was measured.

### 2.4. Data analysis

Flow-sensitive MR perfusion images were obtained by subtraction of the slice-selective and nonselective FAIR acquisitions. Subject motion, assessed by examining the temporal standard deviation images, was deemed negligible. Quantitative analysis of PET images was performed using the two-compartment weighted integration method [34]. No motion correction was performed given the longer acquisition time, due to which the effects of motion are greatly reduced. Three different methods of selecting the ROI were examined. The ROIs were defined on an individual basis due to intersubject slice placement variability. In addition, for group analysis, PET (volume) and fMRI data (single-slice) were resampled into the same reference frame, accounting for PET's lower image resolution.

 (i) *V1-based ROI:* The first ROI selection criterion involved choosing only voxels within the primary visual cortex (V1), since this region should contain the most reliable activation for the stimulus used. V1 was defined using fMRI-based retinotopic mapping with an eccentricity range of 5–10°, as described previously [3, 8, 35], and resampled onto the slice corresponding to the fMRI data for each subject. However, retinotopic V1 regions meeting these criteria can be small, rendering the masking process highly sensitive to misregistration between MRI and PET. Also, a small ROI mask might produce variable results, with activation data outside V1 ignored. Hence, other ROIs types were considered in the analysis.

 (ii) *t-map-based ROI:* The second type of ROI was obtained based on activation *t*-maps for both PET and fMRI CBF images. For fMRI, individual *t*-maps were calculated using *fMRIstat* [36]. PET *t*-maps were automatically generated with the locally developed software used for PET analysis, *DOT* (version 1.8.0, S Milot, MNI) [37]. Both fMRI and PET *t*-maps were thresholded at the 0.05 significance level to obtain the mask, accounting for multiple comparisons for each subject. The overlap between fMRI and PET masks was taken to be the ROI.

(iii) *GM-based ROI:* Since the CBF changes occur mostly in GM, a third set of ROIs, consisting of a GM map in the fMRI occipital lobe slice, was defined for each subject. The GM ROIs were obtained using Bayesian fuzzy classification [38] on the high resolution anatomical MR images. This is well suited for hypercapnia studies, which are best analyzed via the global demarcation of GM. The occipital GM ROIs include activated visual cortical areas.

In this study, the raw FAIR fMRI images have a higher spatial resolution than PET images. To maximize the degree of matching between the MR and PET data, the surface-coil MR anatomical scans were first manually registered to the head-coil images using *Register* (D MacDonald, MNI) [39], then transformed into Talairach space. Prior to the subsequent resampling the surface-coil fMRI data into the head-coil and stereotaxic coordinates, the images were blurred using a 12 × 12 mm^2^ FWHM (full-width-at-half-maximum) Gaussian kernel, resulting in a resolution approximately equal to that of PET data. The postblurring MR data was then resampled into a 1 × 1 mm^2^ grid using trilinear interpolation. Since FAIR data is single slice, PET data was transformed into the FAIR slice space [17]. Individual PET scans were registered to the first PET scan for each subject using an in-house implementation of the variance-of-ratios algorithm [40]. Following this, an average PET scan was calculated for registration of PET onto the MR anatomical space. *DOT* was then used to transform the registered PET images into Talairach space. These images were subsequently resampled onto the same slice from the fMRI data in Talairach space, and the final sampling of the PET CBF slices corresponds to that of fMRI for each subject. All ROI masks were also resampled into Tailarach space, with a resolution of 1 × 1 × 1 mm^3^.

To characterize the relationship between CBF changes measured using PET and FAIR, a correlation analysis was performed. The initial analysis was at the level of individual CBF changes for each subject, averaged across all voxels in the subject's ROIs, while the subsequent analysis was performed on the CBF data averaged across all subjects in the individually defined ROIs.

## 3. RESULTS

### 3.1. Physiological monitoring

Overall, the pulse rate was not influenced by the air composition. It ranged from 55.6 to 58.3 beats/min (subject average) throughout the experiment. The subjects also maintained a reasonably constant respiratory rate, between 15.7 and 17.9 breaths/min. While ETCO_2_ was stable at 40.2–42.1 mmHg during the five normocapnia conditions, a small ETCO_2_ increase of about 4 to 6 mmHg was observed during hypercapnia, in agreement with rise in pressure introduced by CO_2_. Finally, arterial saturation of O_2_ remained constant at 98-99% throughout all the sessions.

### 3.2. Regions of interest

**Figure 3:**
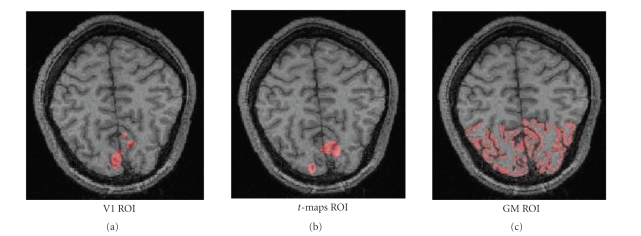
Sample ROIs (shown in red) defined for one subject. The V1 ROI was obtained by retinotopic mapping, the *t*-map ROI included common voxels from thresholded and resampled FAIR and PET *t*-maps, and the grey matter (GM) ROI was defined by Bayesian classification of the anatomical structures.

The three ROIs used for the ΔCBF comparisons were defined on a subject basis, as described in Methods and Materials, and an example is shown in Figure 3. For all subjects, the V1 ROIs were the smallest, containing only between 0.101cc and 0.187cc, due to the stringent retinotopic mapping criteria. V1 was correctly identified by the ROIs, but due to the small mask size, the corresponding ΔCBF measurements are prone to variations introduced by misregistration and motion. On the other hand, the *t*-map ROIs contained between 0.202cc and 0.407cc. To obtain the *t*-map ROI's at *P* = .05, the fMRI *t*-maps were thresholded at 5.42, and the PET maps at 4.45. A lower standard deviation was seen in the responses detected in these ROIs, and the FAIR *t*-maps contained no statistically significant voxels for 2 of the 10 subjects. The largest were the GM ROIs, covering 1.489-0.625 cc for the same group of subjects. Some automatically classified GM voxels were excluded by the certainty-level threshold, but the final GM ROIs were still significantly larger than the V1 ROIs. As previously mentioned, larger ROI masks may include nonactivated voxels, leading to reduced levels of measured ΔCBF. Nonetheless, the GM masks provide an effective means of comparing CBF measurement across modalities, especially for the hypercapnia condition.

### 3.3. Activation time-course

**Figure 4:**
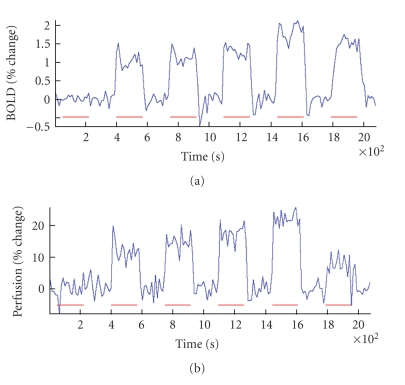
BOLD (a) and FAIR (b) time courses of the CBF percent change in the V1 ROI averaged over 10 subjects are shown, from left to right, corresponding to baseline, 25%, 50%, 75%, and 100% intensity checkerboards and 5% hypercapnia. The horizontal bars represent the 3-minute periods during which the stimulus was on. An increase in signal change can be observed with increasing checkerboard intensity for BOLD and FAIR perfusion, ranging between 1.0%–1.9% and 11.4%–22.5%, respectively.

Time-series CBF data were obtained only from fMRI data as PET measurements were not available as separate frames. The BOLD and FAIR time courses were obtained by averaging all voxels in the ROI and shown in Figure 4 in the V1 ROI of one subject (activation paradigm from left to right: baseline, checkerboards from 25% to 100% intensity and hypercapnia). We observed an increase in the level of signal change with increasing checkerboard intensity for both BOLD and FAIR. As expected, the activation SNR, defined as the ratio of the ROI mean over the standard deviation during activation, was lower for FAIR than for BOLD.

In the averaged time courses, FAIR ΔCBF ranged between 11.4% and 22.5% for the various stimulation intensities, and BOLD changes between 1.0% and 1.9%. These are in the range of percent changes previously reported by Hoge et al. using the same stimulation and acquisition design (between 1.1% and 2.2% for BOLD, and between 23% and 48% for FAIR) [3, 28]. The measured percent changes in small ROIs such as the V1 were expected to decrease with the application of image blurring. This was true for average FAIR ΔCBF in V1, where lower values (between 16.4% and 30.4%) were measured compared to the study by Hoge et al. (where no blurring was applied). Comparatively lower signal changes were also measured during hypercapnia (1.5% and 7.2% for BOLD and FAIR, resp., compared to 2.5% and 21%, resp., in previous studies [3, 8]), despite similarity in observed ETCO_2_ changes. However, our postblurring BOLD percent changes in the V1 ROI (between 1.1% and 2.1%) were still similar to those obtained by Hoge et al.

### 3.4. FAIR versus PET

Relative PET CBF changes were calculated from absolute PET CBF values and compared with relative FAIR ΔCBF changes. PET ΔCBF values in the V1 ROIs for all 10 subjects, as well as averaged ΔCBF over all subjects in the 3 ROI types, are presented in Table 1. The results are in the range of published ΔCBF values [3]. However, the PET ΔCBF measurements have a high standard deviation, largely attributable to the abnormally high ΔCBF measured in subject 8. As previously mentioned, ΔCBF time courses could not be obtained for PET data; instead, time-averaged ΔCBF in the PET ROIs were compared to individual and group-averaged FAIR results. The PET ΔCBF maps were found to have significantly lower SNR than FAIR (<1.0 for V1 and GM ROIs, and <2.27 for *t*-map ROIs) for all the experimental conditions, as seen in Table 1, and to demonstrate considerable intersubject variability under each condition. We further noted that ΔCBF for subject 10 in the V1 and GM ROIs were negative for PET (possibly due to a relatively high PET CBF measured at baseline) and only very slightly positive for fMRI (the subject having no significant voxels in the *t*-maps ROI). Data from this subject is likely to account for much of the standard deviation seen in both techniques.

**Figure 5:**
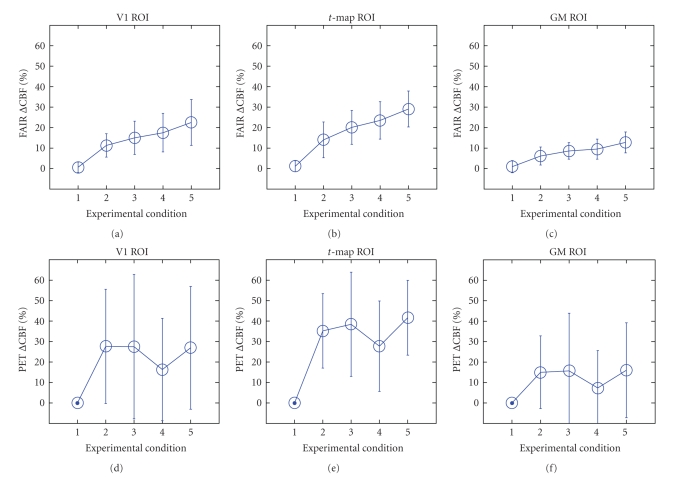
FMRI (top) and PET (bottom) CBF percent changes during visual stimulation in the V1, *t*-map, and GM ROIs. Error bars represent the standard deviation in the data. Experimental conditions 1 to 5: baseline, 25%, 50%, 75%, and 100% intensity checkerboard visual stimulation. The FAIR CBF maps have considerably higher SNRs than PET maps, and there was a consistent monotonic increase in the CBF percent change (ΔCBF) with stimulus intensity in the FAIR data which could not be discerned distinctively in PET given the limited SNR.

**Figure 6:**
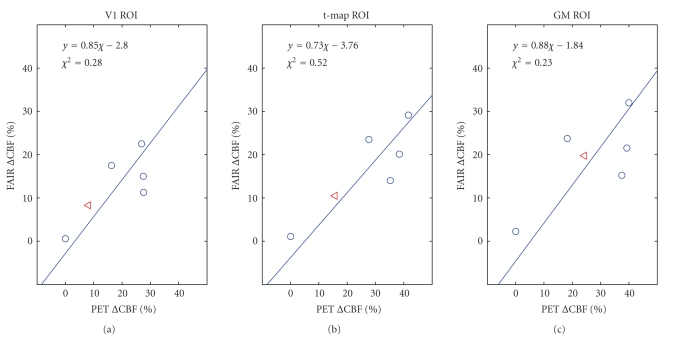
Correlation plots of group-average fMRI CBF percent changes with respect to PET CBF changes in the V1, *t*-map, and GM ROIs. The dots represent results from baseline and 4 levels of visual stimulation, and the triangles represent the results for the 5% hypercapnia condition. The equations resulted from the linear fitting of the data, represented by the dotted lines. The hypercapnic ΔCBF measured using PET and MR are similar. In all 3 cases, the *χ* ^2^ values have probabilities well within the range of acceptance, and *t*-test results indicate the slope of the linear fit is not significantly different from unity.

The ΔCBF values measured with FAIR and PET in all the ROIs during baseline and 4 graded levels of visual stimulation are presented in Figure 5. PET data is shown as having much higher standard deviation than FAIR measurements, even when the GM ROIs were used. The group-averaged results seem to provide a better indication of the CBF changes. Table 2 summarizes the FAIR and PET ΔCBF group averages. The FAIR baseline signal value was obtained from the 6-minute baseline data, whereas in PET, since only one baseline volume was obtained, the baseline percent change was fixed at 0%. The regions of activation-induced ΔCBF in both the V1 and *t*-map ROIs were localized to the expected site of activation. However, as seen in Figure 6, the ΔCBF in the GM ROIs are lower, since many less than maximally activated voxels are likely to have been included in the mask. In addition, a postblurring data resolution of 14 × 14 mm^2^ implies that both GM and WM contribution can be expected in the same voxel; the GM signal intensity thus diminished in both PET and FAIR [3]. We further observed that for all ROIs, increases of FAIR ΔCBF appear to correspond well with increases in visual stimulation intensity, in agreement with previous observations [8]. This was not the case for PET data. Finally, for the hypercapnic condition, ΔCBF in all three ROIs were similar for FAIR and PET.

### 3.5. Correlation analysis

In Figure 6, group-averaged ΔCBF values for baseline and the 4 visual stimulation conditions are shown as dots, while the hypercapnic ΔCBF is represented by a triangle. The hypercapnic ΔCBF values were quite consistent between PET and MR measurements. Note that for all experimental conditions, higher correlation was seen when comparing the PET and MR group averages (Figure 6) than when comparing the two sets of individual subject values (0.76, 0.87, 0.73 versus 0.45, 0.29, 0.57 for the V1, *t*-map and GM ROIs, resp.). The line-fitting algorithm used accounted for data variance on both the PET and the ASL axis [3], and the resulting slopes in all 3 ROI types were similar. *χ* ^2^ values had high probabilities (*q* = 92%–98%), well below the threshold for rejection of the fit (9.488 for 4 degrees of freedom at *P* = .05). Furthermore, the two-tailed *t*-test was used to assess whether the slopes were significantly different from unity. The *t*-values obtained for all 3 ROIs (−0.10, −0.35, and −0.06 for the V1, *t*-map, and GM ROIs, resp.) suggest that the slopes of the MR-PET linear fits do not differ statistically from unity.

## 4. DISCUSSION

Arterial spin labeling (ASL)-based perfusion fMRI techniques are fast, noninvasive, have high resolution (temporal and spatial), and are widely accessible. Furthermore, ASL can easily be used in conjunction with other MR techniques to provide information on a variety of physiological parameters in a single session. The goal of this study was to compare PET and FAIR ASL, under conditions extensively used in numerous brain flow-metabolism studies [3, 25, 26, 41]. The FAIR and PET data were fit to a straight line taking into account the variations in both modalities. The resulting slopes were not significantly different from unity, though large standard deviations were associated with the fit. Furthermore, our results showed a consistent monotonic increase in the CBF percent change (ΔCBF) with stimulus intensity in the FAIR CBF data which could not be observed consistently with PET due to its low SNR. On average, FAIR ΔCBF values were slightly lower than those measured with PET under the same conditions. Also, a lower SNR was observed in the FAIR group-average time course, likely due to intersubject variability and group outliers. Despite these differences, the regional ΔCBF values measured with FAIR were highly correlated with PET measurements.

When comparing PET and fMRI data, a key step was the transformation of both datasets into the same frame of reference and to have the same spatial resolution, since the numerous registration steps could potentially introduce errors. First of all, PET data had to be aligned between runs and also to the surface-coil fMRI data, which was in turn manually registered to the head-coil MR anatomical images through a process susceptible to intersubject variations. Subsequently, PET and MR scans were transformed into Talairach space along with the chosen ROI masks, allowing for additional image degradation. Moreover, since the fMRI data consisted of a single slice, no blurring could be performed in the slice direction, and hence the slice resolution of the FAIR images did not match that of the PET volume data. In some cases where the fMRI slice may have been positioned above the activated area, PET data would reflect activation while the FAIR data may not, resulting in FAIR ΔCBF values being lower than those of PET. This may be one of the sources of the systematic ΔCBF underestimation using this single-slice FAIR implementation. Furthermore, since the scan sessions were usually on different days, measurements are prone to various intrasubject variations. In fact, intrasubject variability as high as 16% has been reported in the literature for trained subjects [12, 14], and simple factors such as caffeine intake can alter the CBF response. Other sources of errors include subject motion and respiration. PET data were acquired with lower temporal resolution and over longer scan periods, thus the effects of motion would be reduced through data averaging. In addition, as all PET scans were aligned to the first run, the effect of potential movement between runs was greatly reduced. Although this process does not account for motion within runs, the need for in-plane motion correction was reduced due to the use of image blurring. On the other hand, each fMRI image was acquired in <100 milliseconds, and motion could have potentially shifted the location of the activated region out of the ROI mask, resulting in an underestimation of the activation as well as diminished SNR. Complete 3D retrospective motion correction was not possible for the MR data, since the surrounding regions were not scanned, but visual examination suggested minimal displacement between runs or between frames in one run. Nonetheless, for both PET and MR, intersession positioning differences could result in a slight data misalignment and therefore comparison of slightly shifted brain regions.

An additional source of potential error could originate from the experimental setup. Although the PET experiments were designed to reproduce the conditions found in the MR environment as closely as possible, some elements in the experimental setup were difficult to reproduce. These include factors such as head and mirror orientations, sensory stimulation induced by MR scanner noise and vibration, the presence of arterial and venous lines during the PET experiment, and the duration of the study. Changes in mirror orientation might occur between subjects and scans. While this should not have a large impact on the activation if the subject maintains fixation on the centre, the quality of the subjects' attention could be influenced by the degree of ease with which the stimulus was viewed. Sensory stimulation from the high background noise and vibration in the MR scanner, absent in the PET environment, could have added an additional variability. This factor was, however, previously reported to have little impact on the analysis [3]. On the other hand, in the PET experiments, the stress associated with having an arterial line and multiple injections could also have affected the subject's response. Moreover, the scan durations for PET and fMRI were different; subject motion generally increases during longer scans. In the fMRI experiments, nearly 36 minutes of continuous stimulation was used with no breaks between runs, whereas in PET, the scan sessions were shorter (4 minutes), and the subjects were given breaks, relieving strain on subject attention.

As we mentioned, the dependence of FAIR CBF estimates on transit delay as well as label width has been previously discussed [3, 19]. The transit delay is influenced by the label gap size, and an underestimation of the delay leads to CBF underestimation. The quantification and correction of the underestimation requires ASL acquisitions at a range of different inversion times for each flow condition. However, such measurements could not be included in this study given the number of graded flow increases being investigated. Instead, in the FAIR implementation used in this study, we made an effort to minimize the influence of transit delays by using a substantially smaller gap size (3 mm) than typically reported in the literature [3, 20]. In addition, we used body coil transmission to achieve a very large label width, thereby minimizing CBF estimation errors due to labeling slab size.

Finally, it should be noted that while the single-slice FAIR sequence we used did not allow the measurement of absolute CBF, the literature has recently described various quantitative and multislice ASL sequences such as Q2TIPS [5] and QUIPSS II with BASSI pulse tagging [21, 42], which would better suit future fMRI studies. Multislice perfusion sequences provide a definite advantage when studying large ROIs, reducing the dependence on slice placement, facilitating visualization of global CBF changes, and benefiting the clinical utility of ASL perfusion imaging. In addition, multichannel acquisitions and higher field strengths can provide higher global SNR while abolishing the need for surface-coil functional scans. This would reduce the number of steps needed for registration of fMRI and PET data by eliminating the subject-dependent manual registration step between head and surface coil anatomical scans. Improved data SNR and reduced postprocessing variability would permit further exploration of ΔCBF variation with stimulation intensity.

In recently published studies involving CBF measurements by our group [22, 23], the QUIPSS II technique was employed [21, 42]. However, as stated earlier, the current study was motivated by the need to assess the accuracy and validity of the FAIR method with its known technical limitations, particularly given the importance attached to results published in recent years based on FAIR measurements [3, 18, 24, 25, 27]. In addition, we wanted to address the intense interest in a comprehensive evaluation of the relative quality of perfusion imaging using MRI and PET, the latter being the de facto golden standard technique for perfusion imaging. The chief finding of the present study is that there was no significant difference between measurements of CBF change using PET and FAIR under matched levels of graded visual stimulation and hypercapnia. These findings directly support the argument that FAIR, while leaving room for improvement, provides a valid measure of CBF changes under our experimental conditions, characterized by an accuracy well within the range measured using H_2_^15^O PET. The other important observation is that CBF measurements made using PET have a much lower SNR than those made using ASL-fMRI, stresses the immense importance in validating the latter for a wide array of applications.

## Figures and Tables

**Table 1:** CBF measured with PET in 10 subjects under 6 experimental conditions (baseline, 4 levels of graded visual checkerboard (CHB) stimulation as well as hypercapnia (HC)) in the V1 ROI, followed by the CBF measurements averaged over the 10 subjects in all 3 ROIs—V1, *t*-map, and GM ROIs. The PET ΔCBF measurements have a high standard deviation, largely attributable to the abnormally high CBF measured in subject 8.

Subject	Baseline	25% CHB	50% CHB	75% CHB	100% CHB	5% HC
mean CBF in V1 ROI for all 10 subjects (mL/100 g tissue/min)						
1	47.7	62.5	50.5	49.0	61.3	46.8
2	34.6	54.4	53.4	43.1	52.4	38.1
3	32.6	58.3	42.6	48.6	55.9	39.8
4	42.4	50.4	55.3	45.1	52.0	60.2
5	36.8	51.6	63.0	39.6	43.4	38.4
6	39.5	46.4	74.7	59.6	62.6	50.7
7	62.5	72.5	62.9	64.3	68.6	65.0
8	114.3	139.5	137.8	164.3	148.7	115.9
9	66.1	80.2	53.6	66.65	76.4	66.6
10	50.0	36.6	45.4	37.1	31.8	35.1

Average mean ± std CBF in VI ROI						
	52.6 ± 24.4	65.2 ± 28.9	63.9 ± 27.6	61.7 ± 37.4	65.3 ± 31.9	55.6 ± 24.1

Average mean ±std CBF in *t*-map ROI						
	53.6 ± 24.5	70.6 ± 27.9	71.3 ± 27.0	68.9 ± 37.6	73.8 ± 30.2	60.0 ± 23.2

Average mean ± std CBF in GM ROI						
	47.0 ± 22.3	52.8 ± 23.2	51.7 ± 20.4	50.3 ± 26.8	52.8 ± 23.9	49.8 ± 21.0

**Table 2:** FAIR and PET CBF percent changes averaged over all subjects (mean ± std), for all conditions (baseline, 4 levels of visual checkboard (CHB) stimulation, and hypercapnia (HC)), in the V1, *t*-map, and GM ROIs. For PET, the baseline percent change was fixed at 0%, since only one baseline volume was acquired.

	Baseline	25% CHB	50% CHB	75% CHB	100% CHB	5% HC
	ΔCBF (%)					
ROI: retinotopically defined VI (mean ± std)						
FAIR	0.6 ± 2.6	11.3 ± 5.7	15.0 ± 8.1	17.5 ± 9.4	22.5 ± 11.2	8.3 ± 6.2
PET	0.0 ± 0.0	27.6 ± 27.9	27.5 ± 35.3	16.3 ± 25.0	26.9 ± 30.0	8.1 ± 19.6

ROI: PET and FAIR *t*-map overlap (mean ± std)						
FAIR	1.1 ± 2.7	14.0 ± 8.7	20.1 ± 8.3	23.5 ± 9.1	29.1 ± 8.8	10.5 ± 7.1
PET	0.0 ± 0.0	35.2 ± 18.2	38.4 ± 25.5	27.7 ± 22.1	41.6 ± 18.3	15.7 ± 18.0

ROI: occipital lobe grey matter (mean ± std)						
FAIR	0.9 ± 3.0	6.1 ± 4.4	8.6 ± 4.0	9.5 ± 4.9	12.8 ± 5.1	7.9 ± 6.2
PET	0.0 ± 0.0	15.0 ± 17.8	15.7 ± 28.2	7.3 ± 18.3	16.0 ± 23.2	9.7 ± 23.0
